# Increased Incidence of Kawasaki Disease in Taiwan in Recent Years: A 15 Years Nationwide Population-Based Cohort Study

**DOI:** 10.3389/fped.2019.00121

**Published:** 2019-03-29

**Authors:** Ying-Hsien Huang, Kuan-Miao Lin, Shu-Chen Ho, Jia-Huei Yan, Mao-Hung Lo, Ho-Chang Kuo

**Affiliations:** ^1^Department of Pediatrics, Kaohsiung Chang Gung Memorial Hospital, Chang Gung University College of Medicine, Kaohsiung, Taiwan; ^2^Kawasaki Disease Center, Kaohsiung Chang Gung Memorial Hospital, Kaohsiung, Taiwan; ^3^Department of Pediatrics, Chiayi Chang Gung Memorial Hospital, Chiayi, Taiwan; ^4^Department of Public Health, College of Health Sciences, Kaohsiung Medical University, Kaohsiung, Taiwan

**Keywords:** Kawasaki disease, incidence, cohort study, Taiwan's National Health Insurance Research Database, seasonality

## Abstract

**Background:** Kawasaki disease (KD) is diagnosed in children suffering from fever for more than five days and five clinical characteristic symptoms. The aim of this article was to research the clinical characteristics among KD children in Taiwan in recent years through a population-based cohort study.

**Materials and Methods:** We carried out a nationwide retrospective cohort study by analyzing the data of KD patients (ICD-9-CM code 4461) from Taiwan's National Health Insurance Research Database (NHIRD) during the period of 1996-2011.

**Results:** Among all the insured children in the NHIRD, insurance claims data were reported for 13,260 patients diagnosed with KD, with 8394 (63.30%) subjects being administered IVIG for treatment. Of the patients diagnosed with KD, 94% were under the age of 5 years old, and the majority of cases occurred in May. Furthermore, the incidence of KD more than doubled (28.58–60.08 per 100,000) during this period in Taiwan.

**Conclusion:** We developed a five-based mnemonic device for parents and first-line clinicians to easily use in order to diagnose KD. We also observed an increased incidence of KD in Taiwan during the study period. In addition, we develop a five-based mnemonic device for parents and first-line clinicians in clinical diagnosis of KD can easily remember: Fever> 5 days, 5 clinical criteria, predominantly in children <5 years of age, and peak seasonal clustering in the 5th month, May (April–June) in Taiwan.

## Introduction

Kawasaki disease (KD) is an acute febrile vasculitis syndrome that affects various systems, has an unknown etiology, and generally occurs in children under the age of 5 years old (1). KD is diagnosed in children who have a prolonged fever for more than 5 days, and five major clinical signs (2). The vascular involvement of KD occurs in both small and medium-sized blood vessels, particularly the coronary arteries (3). The most serious complication of KD is a coronary artery lesion (CAL), including myocardial infarction, and coronary artery aneurysms (CAA) (4). Approximately 20% of untreated children developed a sequelae of vasculitis with a coronary artery aneurysm (5). A U.S. multicenter study determined that a single high-dose of 2 g/kg intravenous immunoglobulin (IVIG) plus aspirin can lower the incidence of CAA from 20–25% to 3–5%, as well as reduce fever duration (6).

The global prevalence of KD in children is highest (218/10^5^) in Japan and the lowest (4.7/10^5^) in children of European descent, while Taiwan has an incidence of 66/10^5^ (2). However, a larger population-based cohort study is required to confirm these observations and determine the clinical characteristics of KD children. Therefore, in the present study, we aimed to examine both the incidence and clinical characteristics of KD patients in a nationwide KD dataset obtained from the health care database of Taiwan's National Health Insurance (NHI).

## Methods

In this study, we used data from the medical claims database of Taiwan's National Health Institute (NHI) program. Implemented in Taiwan on March 1, 1995, the NHI program provides compulsory universal health insurance and has information about nearly 99% of the 23.74 million residents of Taiwan based on ethical democratic principles (7). Previous studies have described in detail the National Health Insurance Research Database (NHIRD) (8, 9), which contains such medical information as insured individuals' inpatient and outpatient care facilities, prescriptions, gender, date of birth, date of visit or hospitalization, and diagnosis. All diagnoses are coded using the International Classification of Diseases, Ninth Revision, Clinical Modification (ICD-9-CM) format. Information from the NHIRD database has been shown to be complete, reliable, and accurate for use in epidemiological studies (7).

Children diagnosed with KD (ICD-9: 4461) under the age of 20 years old include untreated KD, KD with aspirin administration, and KD with IVIG administration. Coronary artery aneurysm (CAA) was coded as 414.11 in the ICD-9-CM. We defined KD recurrence as a period of >1 month between two hospitalizations.

We identified and enrolled a cohort of 13,260 KD patients between January 1997 and December 2011 in this study. All patients were categorized into one of the following three groups: ICD-9+Aspirin, which was defined as patients with two visits within 60 days that received aspirin therapy; ICD-9+IVIG, which was defined as inpatients that received IVIG treatment at diagnosis; and ICD-9 without IVIG, which was defined as inpatients that did not receive IVIG or aspirin treatment. The ICD-9+IVIG group was a specific indicator of KD in the acute stage, while the ICD-9+Aspirin group was a specific indicator of KD in the afebrile stage upon diagnosis. The present study was exempt from full review by Chang Gung Memorial Hospital's Institution Review Board (IRB No.102-0364B) because the patients' identification numbers in the database were encrypted to protect their privacy (10).

### Statistical Analysis

The differences between these three KD groups of sex, age group (<1, ≥1~ <2, ≥2~ <3, ≥3~ <4, ≥4~ <5, and ≥5 years-old), and CAA were analyzed by the Pearson chi-square test. The year-specific incidence rates (per 1000 person-y) of KD were calculated by dividing the number of newly diagnosed KD by the total number of children below 5 years of age in Taiwan each year. The annual incidence rate of KD was calculated as the number of KD per 100,000 children <5 years of age. We performed all analyses using the SAS statistical package (version 9.3; SAS Institute Inc., Cary, NC, USA). Significance was assigned for *p* < 0.05.

## Results

### Clinical Characteristic of KD

The purpose of this study was to analyze the general characteristics and annual incidence of KD by collecting patients' data from the ICD-9+IVIG and ICD-9+Aspirin groups. The study participants' general characteristics are summarized in Table 1. We analyzed 13,260 children diagnosed with KD (ICD-9) under 20 years old in the period between January 1997 and December 2011. Of those, 4614 (35%) were categorized as KD alone (ICD-9), 8394 (63%) as KD with IVIG administration (ICD-9+ IVIG), and 252 (2%) as KD with only aspirin administration (ICD-9+ Aspirin). Of the 8646 patients in the ICD-9+IVIG and ICD-9+ Aspirin groups, the male-to-female ratio was approximately 1.32 (4918/3728), and 8113 (94%) were <5 years old. The incidence of coronary artery aneurysm (CAA) was 10–12%, with a recurrence rate of 2% in ICD-9+ IVIG and ICD-9+ Aspirin groups and 8% with a recurrence rate of 1% in ICD-9+ IVIG and ICD-9 alone groups. Interestingly, patients in the ICD-9 group without IVIG or aspirin treatment demonstrated no significant gender difference (male-to-female ratio = 1.03), which may indicate that this group was not in the acute stage, was not used for the coronary arteries survey, or was the result of overdiagnosis or misdiagnosis (11).

**Table 1:** General characteristics of study participants.

	**Total**	**ICD-9+aspirin**	**ICD-9+IVIG**	**ICD-9**	***p-value***
Number of (%) patients	13,260	252 (2)	8,394 (63)	4,614 (35)	
**GENDER**					
Male	7,264 (55)	156 (62)	4,762 (57)	2,346 (51)	<0.0001
Female	5,996 (45)	96 (38)	3,632 (43)	2,268 (49)	
**AGE GROUP (%)**					
0~ <1 y	4,727 (36)	63 (25)	3,117 (37)	1,547 (34)	<0.0001
≥1~ <2 y	3,777 (28)	58 (23)	2,504 (30)	1,215 (26)	
≥2~ <3 y	1,928 (15)	48 (19)	1,220 (15)	660 (14)	
≥3~ <4 y	1,046 (8)	26 (10)	635 (8)	385 (8)	
≥4~ <5 y	700 (5)	21 (8)	421 (5)	258 (6)	
≥5 y	1,082 (8)	36 (14)	497 (6)	549 (12)	
**CAA (%)**					
Without	12,083 (91)	221 (88)	7,593 (90)	4,269 (93)	<0.0001
With	1,177 (9)	31 (12)	801 (10)	345 (8)	
Recurrent	174 (1)	5 (2)	126 (2)	43 (1)	0.0154

### The Seasonality of Kawasaki Disease in Taiwan

**Figure 1:**
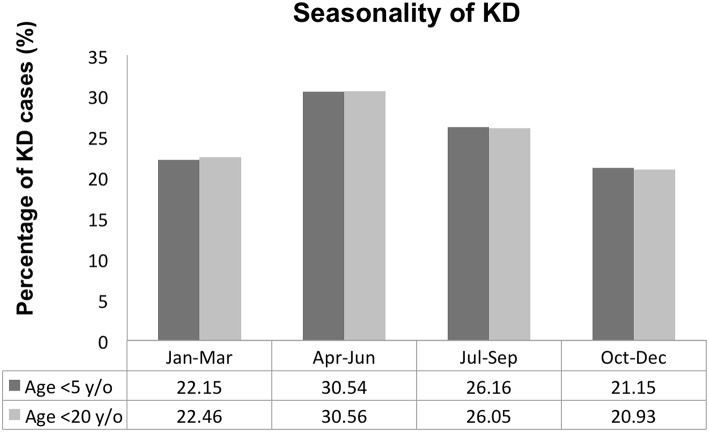
The seasonality of Kawasaki disease in Taiwan.

Table 2 shows KD patient distributions by month. In the ICD-9 + IVIG group, the peak months of distribution were April-June, while patient distributions decreased in the winter months (November–February). The monthly distribution trends were similar in children <5 years old (Table 3, Figure 1). The annual incidence rates per 100,000 children <5 years old are shown in Table 4.

**Table 2:** Patient distribution by month of study participants under the age of 20 years old.

**Month**	**Total**		**ICD-9+aspirin**		**ICD-9+IVIG**		**ICD-9**	
	***N***	**%**	***N***	**%**	***N***	**%**	***N***	**%**
January	874	7	16	6	539	6	319	7
February	945	7	22	9	584	7	339	7
March	1,154	9	28	11	762	9	364	8
April	1,233	9	33	13	784	9	416	9
May	1,357	11	20	8	881	11	456	10
June	1,300	10	23	9	900	11	377	8
July	1,264	10	23	9	766	9	475	10
August	1,183	9	16	6	700	8	467	10
September	1,127	9	21	8	721	9	385	8
October	1,050	8	15	6	663	8	372	8
November	934	7	16	6	586	7	332	7
December	839	7	19	8	508	6	312	7

**Table 3:** Patient distribution by month of study participants under the age of 5 years old.

**Month**	**Total**		**ICD-9+aspirin**		**ICD-9+IVIG**		**ICD-9**	
	***N***	**%**	***N***	**%**	***N***	**%**	***N***	**%**
January	793	7	13	6	501	6	279	7
February	829	7	18	8	534	7	277	7
March	1,056	99	25	12	714	9	317	8
April	1,133	9	26	12	733	9	374	9
May	1,236	10	17	8	819	10	400	10
June	1,213	10	20	9	860	11	333	8
July	1,163	10	20	9	722	9	421	10
August	1,080	9	14	6	666	8	400	10
September	1,046	9	20	9	678	9	348	9
October	981	8	15	7	626	8	340	8
November	867	7	13	6	558	7	296	7
December	781	6	15	7	486	6	280	7

**Table 4:** Incidence of study participants under the age of 5 years old.

**Year**	**Population under 5 y**	**Total**		**ICD-9+aspirin**		**ICD-9+IVIG**		**ICD-9**	
		**Case**	**Incidence**	**Case**	**Incidence**	**Case**	**Incidence**	**Case**	**Incidence**
1997	1599094	766	48	12	1	457	29	297	19
1998	1545889	983	64	8	1	569	37	406	26
1999	1507221	903	60	15	1	526	35	362	24
2000	1489242	907	61	11	1	515	35	381	26
2001	1426759	964	68	20	1	632	44	312	22
2002	1350829	838	62	13	1	387	30	438	32
2003	1309903	737	56	14	1	395	30	328	25
2004	1243939	800	64	9	1	406	32	385	31
2005	1144355	807	71	25	2	579	51	203	18
2006	1092942	791	72	16	1	620	57	155	14
2007	1052585	718	68	9	1	528	50	181	17
2008	1026206	803	78	18	2	602	59	183	18
2009	1002160	679	68	15	2	484	49	180	18
2010	964093	767	80	13	1	622	65	132	14
2011	956990	715	75	18	2	575	60	122	13

The increased incidence of KD in recent years in Taiwan

**Figure 2:**
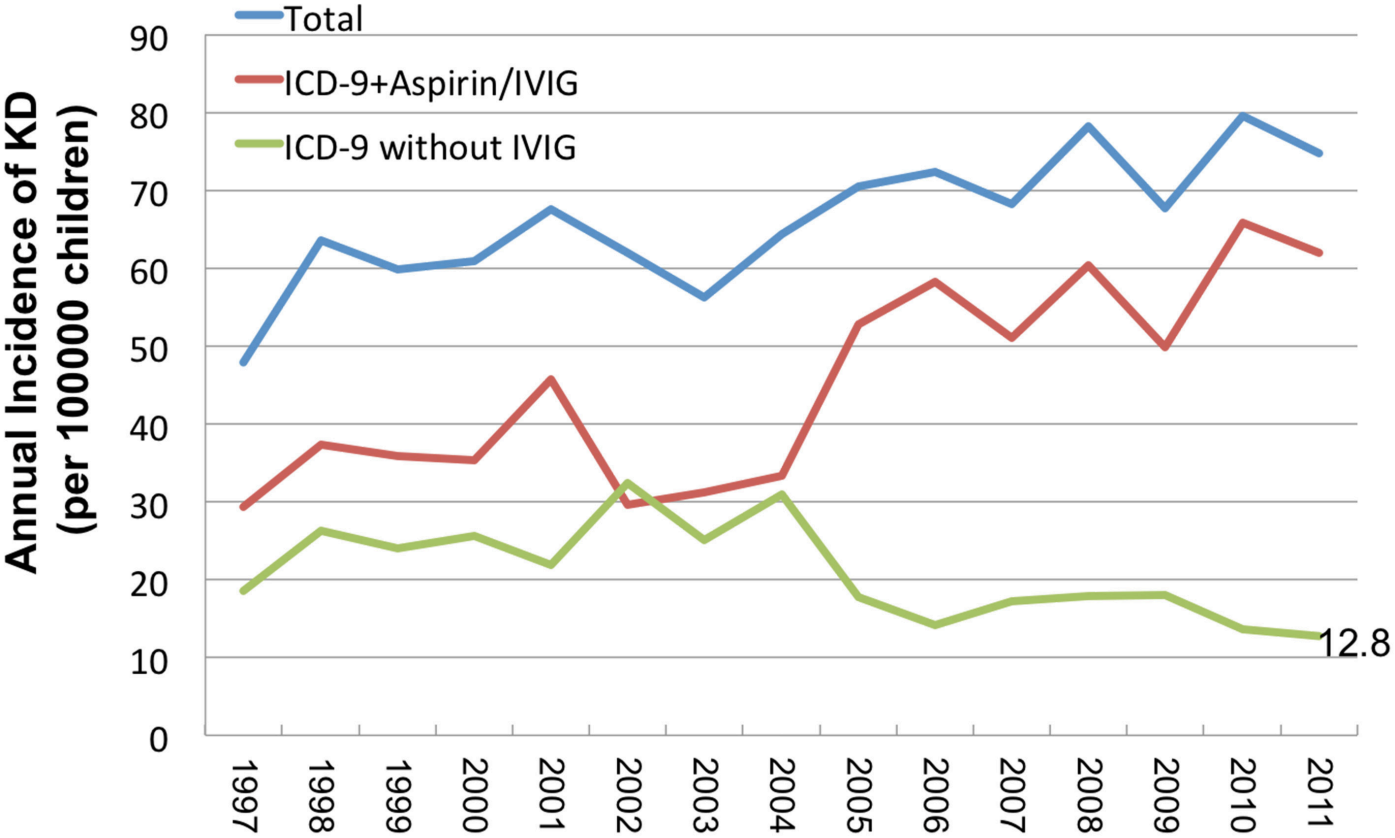
The increased incidence of KD in recent years in Taiwan.

Since the ICD-9+ IVIG and ICD-9+Aspirin groups were diagnosed with KD more accurately in Taiwan's NHI health care database, we found that the incidence ranged from 29.3 to 62.0 per 100,000 children from 1997 to 2011 among these patients. The average incidence from 1997 to 2011 was 45.2 per 100,000 children under the age of 5 years old. Overall, the incidence of KD in Taiwan has significantly increased in recent years. We also found that the incidences decreased from 18.6 to 12.8 per 100,000 children from 1997 to 2011 in the ICD-9 alone group, indicating that both the increased accuracy of KD diagnosis and the incidence discrepancy between the two groups were also significant (Figure 2).

## Discussion

Today, KD is commonly diagnosed and reported in global populations, especially in Asia (12). We have observed that the incidence of KD has increased in recent years in Taiwan, as well as in Beijing and Japan (13, 14). Determining whether this observation is the result of increased diagnosis due to improved awareness and/or access to specialist medical services or reflective of an actual increase in incidence is difficult (15). Both factors likely play a role in many countries, particularly in those countries where KD has been described more recently (16).

With regard to age, KD generally occurs in children <5 years old, which represents the turning point of the host's immune maturation (15). Therefore, KD may be associated with the maturing immune system of young children (11). Furthermore, with regard to gender, our study showed that KD occurs more commonly in the male gender (17). Overall, KD incidence has increased considerably (18) with the increased incidence of allergic diseases worldwide (19). In previous studies, we also discovered that children with KD were at a higher risk of developing atopic dermatitis during the 5 year follow-up period than the control group (10), as well as for allergic diseases in a population-based matched cohort study (8).

According to pathologic studies and climatologic studies, the seasonality of KD may be associated with such triggers as environmental factors or infectious pathogens (20). Our results indicate that the peak months of KD in Taiwan are April, May, and June, which is in line with a previous study that observed an increased incidence of KD in Taiwan during the summer (21). However, Japan and Korea have been found to have two peaks of seasonal incidence- winter and summer, while the peak incidence of KD in China was observed as spring and summer (22), with results that resemble those of our study. A global prospective study found that KD case numbers were greater in January through March in the extra-tropic region of the Northern Hemisphere but showed weak significance in seasonal variation in the tropic and the Southern Hemisphere extra-tropics region due to scarce data collection (23). To explain seasonal variations, Abrams et al. reviewed KD cases from 1991 to 2004 in Japan and suggested that the increased incidence was associated with higher precipitation and lower temperatures in the 2 months prior to disease onset (24). The hypothesized “tropospheric wind pattern” mechanism helps explain the three major KD epidemic occurrences in Japan, which occurred in 1979, 1982, and 1986. Said seasonal wind may bring the supposed KD agent across the Northern Hemisphere and has allowed scientists to observe the relationship to KD seasonality (25).

The key to preventing CAL in KD patients is timing the diagnosis and administration of IVIG. In our study, we combined clinical presentations with KD characteristics to develop a five-based mnemonic device for KD that parents and first-line clinicians in clinical diagnosis of KD can easily remember: Fever >5 days, 5 clinical criteria, predominantly in children <5 years of age, and peak seasonal clustering in the 5th month, May (April–June). Clinical diagnosis criteria (Kuo Mnemonic: 1-2-3-4-5) include diffuse mucosal inflammation with strawberry tongue and fissure lips (1 mouth), bilateral non-purulent conjunctivitis (2 eyes), unilateral cervical lymphadenopathy (3 fingers check lymph node), indurative angioedema over the hands and feet (4 limbs), dysmorphic skin rashes (5 or more skin rashes) as our previous report (3).

Our study has some limitations that should be mentioned at this point. First, our study was a single-country study, but the seasonality of epidemiology should be discussed throughout the climate zone. Second, the climate has changed considerably in recent years due to global warming, which may affect seasonal clustering. Furthermore, we did not perform an in-depth analysis of atypical temperature changes, which may have increased the incidence in other months in recent years. Third, data from years ago may under-estimate the incidence of KD due to clinicians' insufficient education or pediatric medication being less popularized. In the future, we need additional worldwide studies to analyze the epidemiology and seasonal effects of KD.

## Conclusion

We have observed an increased incidence of KD in recent years, with a higher incidence in children under the age of 5 years old and a seasonal peak during April–June. Based on our clinical presentation, we developed a five-based mnemonic for KD that parents and first-line clinicians in the clinical diagnosis of KD can easily remember: Fever >5 days, 5 clinical criteria, predominantly in children <5 years of age, and peak seasonal clustering in the 5th month, May (April–June) in Taiwan.

## Author Contributions

Y-HH and K-ML wrote the manuscript. S-CH calculated data. J-HY and M-HL collected data. H-CK final proof check before submission.

### Conflict of Interest Statement

The authors declare that the research was conducted in the absence of any commercial or financial relationships that could be construed as a potential conflict of interest.
